# Air Pollution-Related Health Impacts on Individuals Experiencing Homelessness: Environmental Justice and Health Vulnerability in Salt Lake County, Utah

**DOI:** 10.3390/ijerph17228413

**Published:** 2020-11-13

**Authors:** Angelina L. DeMarco, Rebecca Hardenbrook, Jeff Rose, Daniel L. Mendoza

**Affiliations:** 1 Department of Anthropology, University of Utah, Salt Lake City, UT 84112, USA; angelina.demarco@anthro.utah.edu; 2 Department of Mathematics, University of Utah, Salt Lake City, UT 84112, USA; rebeccah@math.utah.edu; 3 Department of Parks, Recreation, and Tourism, University of Utah, Salt Lake City, UT 84112, USA; jeff.rose@utah.edu; 4 Department of City & Metropolitan Planning, University of Utah, Salt Lake City, UT 84112, USA; 5 Department of Atmospheric Sciences, University of Utah, Salt Lake City, UT 84112, USA

**Keywords:** air pollution, environmental justice, chronic homelessness, unsheltered homelessness, marginalized populations, hidden populations

## Abstract

Experiences of homelessness, although widely varied, are characterized by extensive time in public spaces, often outdoors. However, there has been little empirical research about the ways in which environmental factors affect individuals experiencing homelessness (IEHs). Therefore, the purpose of this study was to use an environmental justice approach to understand how cardiopulmonary health of IEHs is affected by episodic poor air quality in Salt Lake County. It was hypothesized that people who had experienced unsheltered homelessness and those who had been experiencing homelessness for longer periods of time would report greater health difficulties from poor air quality exposure. Through a combination of in-person semistructured interviews with IEHs (*n* = 138) and access to corresponding state-based service provider databases, researchers examined both overall descriptives of and relationships between types (sheltered and unsheltered) and duration (chronic and nonchronic) of homelessness. More than 61% of IEHs reported physical reactions to air pollution, 37% reported air pollution-related emotional stress, and more than 89% had sought medical attention for a condition related to air pollution. Findings indicate that while IEHs report a number of health effects related to poor air quality, there were no significant differences between individuals based on either sheltered status or duration of their experiences of homelessness. This study provides an initial empirical inquiry to understand how environmental disamenities negatively influence IEHs, as well as noting that sheltered status and duration of homelessness are less impactful than originally hypothesized.

## 1. Introduction

### 1.1. Motivation

Exposure to air pollution worsens individuals’ health by increasing cardiovascular and pulmonary events [1,2,3], exacerbations of asthma [4], and mortality [5,6]. Fine particulate matter (PM_2.5_) and ozone, even low levels of exposure, have resulting in increased rates of mortality [7]. Measures to curb these emissions have improved health outcomes [8]. Several studies have focused on the impact of environmental hazards on children, with specific additional emphasis on environmental justice [9,10,11]. Associated health impacts are intensified for vulnerable individuals, such as those experiencing homelessness. Currently, there is a significant lack in research regarding the differential exposure experienced by vulnerable and at-risk groups, specifically individuals experiencing homelessness. The specific aim for this study was to test for disparities in exposure to air pollution among the population of individuals experiencing both sheltered and unsheltered homelessness in Salt Lake County, and to understand how the duration of homelessness affected a variety of health outcomes. In this paper, we provide background literature concerning various experiences of homelessness and how these experiences may be affected by negative air quality. After describing the methodological techniques, we present results from interviews with 138 individuals experiencing homelessness (IEHs). We conclude by discussing the ways in which these results might affect the literature base and our current understandings of homelessness management.

### 1.2. Literature Review

Contemporary homeless populations are diverse and growing. In the United States, homeless populations include individuals often found on the margins of society, as well as those facing extreme poverty, mental illness, and temporary or long-term unemployment. Military veterans, individuals with disabilities, youth, runaways, individuals with drug dependency, itinerants, immigrants, and prostitutes are disproportionally represented in this population [12]. The US Department of Housing and Urban Development (HUD) defines homelessness as a person who “lacks a fixed, regular, and adequate nighttime residence” and clarifies that the person is in one of the following two categories: (a) sleeping in a public or private place not meant for human habitation (unsheltered) or (b) living in a publicly or privately operated temporary shelter (sheltered). While there are multiple categories of institutions that are considered part of the sheltered status definition (e.g., emergency shelters, congregate shelters, transitional housing, motels paid for by charitable organizations), unsheltered homelessness may include people living in spaces including cars, parks, abandoned buildings, condemned buildings, greenfields, brownfields, streets, alleyways, and others [13]. For many of these people, with social services either unavailable, inaccessible, or undesirable, individuals facing unsheltered homelessness turn to public spaces for meeting basic human needs such as eating, sleeping, socializing, urinating, defecating, and other necessities of life [14]. With these necessities, and the ability to meet them, in question, individuals facing homelessness are some of the most vulnerable in society, and face increased health concerns [15,16]. Additionally, while individual experiences vary widely, IEHs often exhibit low health literacy and health-related self-care [17]. Recent estimates indicate that on any given evening, there were more than 567,000 people experiencing homelessness in the US in 2019, and more than 37% of these people were experiencing unsheltered homelessness [18]. People experiencing unsheltered homelessness or in crisis accommodation tend to exhibit the highest levels of psychological distress [19] and are more exposed to environmental health concerns [20]. Finally, research shows that in addition to the expected differences in health outcomes between sheltered and unsheltered individuals, the duration of homelessness matters. A person is considered “chronically homeless” if they have experienced homelessness for at least a year, while also living through a disabling condition such as serious mental illness, substance use disorders, or a physical disability [21]. While all experiences of homelessness increase health concerns, people experiencing chronic homelessness are particularly vulnerable to concerns about both health and personal safety [22], even if levels of psychological distress tend to decline over time as homeless duration increases [19].

While homelessness has long been a concern of sociological, psychological, epidemiological, economic, and health researchers, it has only recently been explored as a topic of environmental justice [23,24]. Environmental justice research and scholarship contends that exposures to pollution and other environmental risks are unequally distributed by a variety of social markers, with particular emphasis on race and class [25]. Environmental justice work has traditionally examined marginalized and vulnerable communities’ exposures to particular hazards, and IEHs regularly live, work, sleep, and exist in increasingly exposed physical locations, where basic biophysical functioning is often difficult and contested [14]. Providing environmentally just conditions like clean water and clean air in those places is critical, but that people have adequate access to these spaces and resources must also be a priority [26]. Everyday experiences are often contested, and, thus, IEHs are threatened with violence at the hands of the criminal justice system. Simultaneously, and increasingly for those facing unsheltered homelessness, difficult environmental conditions associated with everything from basic seasonality to extreme weather events and disasters can have dire implications for health and resilience [16,27,28,29]. However, recent critical work illustrates that sociopolitical experiences (e.g., criminalization, eviction, stigmatization, and marginalization) are often antecedent causal factors in IEH’s spatial displacement and being pushed further into toxic spaces [23]. More pointedly, IEHs are often positioned as an environmental problem to be solved, in turn dehumanizing individuals themselves as a supposed environmental disamenity [26,30]. Resistance to such measures may lead to further threat of eviction and displacement, fueling an iterative cycle of political marginalization, criminalization, and hazard exposure, which ultimately leads to both increased municipal costs and frustrations from housed and unhoused residents alike [31]. Consonant with procedural justice, IEHs, when asked about various environmental injustices, pointed to local municipal decision-making processes as fundamental areas of disenfranchisement [32]. Further, homelessness has emerged as a central concern of scholars of environmental and public health [33,34], demonstrating that individuals facing both sheltered and unsheltered homelessness fundamentally struggle with structural and individual concerns in achieving stable housing situations.

Despite the growing consideration of homelessness within these fields, there is little empirical evidence concerning the environmental disamenities that IEHs face, including how populations effectively living and operating outside in urban environments deal with issues of degraded air quality. Specifically, in Salt Lake County, it was found that during winter inversions the western side of the Salt Lake Valley experiences higher levels of pollutants [35,36]. These studies have highlighted the disproportionate effects of poor air quality on certain vulnerable populations, which lead to this particular research question: how are IEHs affected by poor air quality in Salt Lake County?

### 1.3. Study Description

There is a significant gap in the literature concerning the physical and mental health effects of environmental disamenities (e.g., disproportionate impacts of environmental hazards) like air pollution on IEHs. There has yet to be any empirical data or speculative inquiry into how IEHs interact with the air pollution in which they work, sleep, and live. Therefore, this research project uses an environmental justice approach to provide an initial empirical inquiry into what amounts to the first examination of how poor air quality impacts different subpopulations of homelessness. We contend that environmental justice activists and scholarly movements should engage more deeply and systematically with experiences of homelessness, and expand our research efforts to include more environmental disamenities to understand how they are experienced differentially across housing status, both in the US and elsewhere.

## 2. Materials and Methods

The aim of this research project was to understand IEHs interactions with poor air quality, as well as to determine if a relationship between air pollution and health outcomes existed among this population. Further, we sought to understand if health outcomes were impacted by an IEH’s shelter status (e.g., sheltered or unsheltered) and duration of homelessness (e.g., chronic or nonchronic, experiencing more or less than 52 weeks of homelessness). We sought to determine the statistical relationships between episodic air pollution events and cardiopulmonary health effects on this vulnerable population. Our research begins to fill an empirical gap concerning IEHs and experiences of air quality through an analysis of both in-person qualitative interviews of IEHs in Salt Lake County, and health data from the state of Utah’s Homeless Management Information System (HMIS) database [37].

### 2.1. Data Collection

Researchers conducted on-site interviews with IEHs. The interviews were conducted with both individuals currently residing within emergency shelters and unsheltered individuals primarily residing outdoors. Inclusion criteria required that individuals were experiencing sheltered or unsheltered homelessness, willing to provide their full name and date of birth, and willing to release their information documented within the HMIS system. Only one individual declined to participate in the study as they were unwilling to sign the consent form. Study participants were members of a number of subpopulations. First, interviews were conducted with individuals experiencing sheltered homelessness. These participants were residing at one of two local gender-specific resource centers, designed to house either women or men. Secondly, researchers worked with local social service providers who offer “street outreach” services to individuals experiencing unsheltered homelessness. In these settings, researchers conducted on-site interviews with both women and men. In these unsheltered situations, individuals were living in conditions that were beyond formal homelessness shelters, including locations deemed unsuitable for human habitation. These locations often include sleeping in encampments within tents or makeshift shelters, in abandoned buildings, in sleeping bags in city parks or along streets, and in parking structures or under highway overpasses. Individuals that we encountered spent their days outside of the local library, walking around downtown, in day centers, or remaining within their tent-based encampments.

Semistructured interviews were utilized in an effort to obtain both quantitative and qualitative data. The interview tool obtained quantitative data in the form of self-disclosed demographic, behavioral, and health information for each research participant. The tool included open-ended questions that allowed participants to provide detailed qualitative descriptions about their experiences with air pollution, as well as providing normative information to establish baseline data. The interviews focused on individuals’ sheltered status, duration of homelessness, experiences within the healthcare system, personal health struggles, and experiences with poor air quality while experiencing homelessness in Salt Lake County. More specifically, health outcomes collected during interviews included self-reports of IEH’s visitation patterns to medical providers due to air pollution-related health concerns, whether participants experienced difficulty breathing, experienced headaches, and experienced mental health illnesses. Seeking medical attention for air pollution-related complaints is directly related to whether an individual’s health is impacted by poor air quality. Difficulty breathing and headache data points were collected as they are representative of common cardiopulmonary symptoms that are easily self-reported. Research has linked mental health problems with increased exposure to air pollution, a negative health outcome of poor air quality [38,39,40,41]. In addition to the standard semistructured interviews, an extended interview with a smaller number of participants included more specific questions regarding cardiopulmonary illnesses and other medical concerns.

On-site, in-person interviews with individuals residing in resource centers (i.e., temporary sleeping shelters for IEHs) were conducted in the dining area and all individuals that were encountered were offered the opportunity to participate. Interviews with individuals experiencing unsheltered homelessness (i.e., residing outdoors in encampments or otherwise) were conducted in publicly accessible spaces often chosen by the participants. These locations included outside the local library, in a winter warming center (i.e., “day shelter”), and on the streets of downtown Salt Lake City, with all individuals encountered given the opportunity to participate. All interviews were conducted one-on-one and in seclusion where possible, in an attempt to provide privacy and encourage individuals to answer questions without influence. Interviews were not digitally recorded but recorded on detailed paper forms that included check boxes for binary questions and space for writing out responses to qualitative, open-ended questions. Researchers made all attempts to create safe environments for participants, as these individuals are members of a vulnerable population, and much of the information they provided was deeply personal. Most individuals that were encountered were willing and excited to participate. They were interested in the research and often quite emphatic in their responses. Additionally, participants often helped us recruit other individuals to take part in the study.

Finally, further data from HMIS was also obtained for the majority of research participants. Utah’s HMIS is a state database that collects and stores individual-level interactions that IEHs have with state-sponsored service providers. Individual records include assessments and enrollments (e.g., demographic, health, and time experiencing homelessness information) and service interactions (e.g., resource centers, emergency shelters, motel vouchers, day centers, warming centers, and street outreach). These data from HMIS were used to supplement and extend the analyses of the interview data. While the interview data might be seen as subjective, self-reported data, information from the HMIS database might then be seen as a more external, objective measure of particular aspects of the experiences of homelessness. Included in the analyses of the HMIS database were the number of total and recent nights spent in emergency shelter (sheltered), number of days spent in day-shelters/warming centers (unsheltered), and interactions with institutionalized street outreach efforts. These data provided a measure from an institutional perspective of the number of each individual’s sheltered and unsheltered nights. Sheltered time included the number of nights spent within an emergency shelter and nights spent utilizing shelter-sponsored motel vouchers. Unsheltered time included the number of days that an individual spent in a day shelter or warming center and documented time residing outdoors from street outreach providers. Instances where an individual spent time in a day shelter or warming center and emergency shelter within the same 24-h period, were classified as time sheltered.

### 2.2. Data Analysis

Qualitative data from IEHs were analyzed using a thematic-based approach. In such an approach, open-ended questions were reviewed by multiple researchers, and themes were identified based on commonalities within the data [42]. For questions regarding physical symptoms or responses to air pollution, common responses included chest discomfort, headaches, difficulty breathing, itchy eyes and throats; these responses were then grouped together based on the type or location of the physical ailment. When identifying air pollution being present in the air, common responses were based on the senses (e.g., taste, smell, and sight), with responses again being grouped together based on the common theme (i.e., sense). A codebook was created for each identified theme, with detailed descriptions for each code used within the dataset. The qualitative data were then recoded, using the codebook created by the research team. The qualitative data were analyzed to provide descriptive quantitative statistics so that researchers could more clearly understand common perceptions and health outcomes of the study population.

### 2.3. Research Ethics

Ethical approval for this research was obtained from the authors’ affiliated university prior to the study (IRB_00124147), as well as approval from Utah’s statewide HMIS board, which also was covered by the aforementioned Institutional Review Board (IRB) document. Prior to beginning each interview, participants were consented and signed a release of information for HMIS. Individuals residing within the resource centers and individuals residing outside were approached and invited to participate in the research study; participants were subsequently provided with a small gift card to a local grocery store after completing the interview.

## 3. Results

This section provides an overview of the descriptive statistics derived from the on-site qualitative interviews, with the intended outcome of better understanding the role and influence of a particular environmental disamenity (air quality) on individuals experiencing varying types of experiences of homelessness. The results provide detailed engagement with IEHs perspectives on the health effects of episodic air pollution events.

### 3.1. Study Sample and Basic Demographics

The demographics and characteristics of the study participants were wide-ranging. Standard and extended semistructured interviews were conducted in both women’s and men’s homeless resource centers, as well as with individuals currently experiencing unsheltered homelessness. Overall, semistructured interviews were conducted with 138 IEHs, including 120 standard interviews and 18 extended interviews. We interviewed 57 females and 81 males, with a mean age of 46.46 years (see Table 1). At the time of the interview, 70 individuals were sheltered and 68 individuals were unsheltered. Researchers obtained interviews from 35 women and 35 men staying within the resource centers. Interviews were conducted in shelters designed to temporarily house women (*n* = 5 extended, and *n* = 30 standard) or men (*n* = 5 extended and *n* = 30 standard). Researchers worked with local social services providers that offer “street outreach” services to individuals experiencing unsheltered homelessness to obtain surveys from both women (*n* = 4 extended and *n* = 12 standard) and men (*n* = 4 extended and *n* = 48 standard) living in locations deemed unsuitable for human habitation. Across these 138 interviews, each lasting an average of approximately 17 min, a substantial amount of qualitative data was collected, totaling more than 40 h of in-person, on-site interviews. Finally, additional data from HMIS was obtained for 130 of the possible 138 research participants, with eight individuals unable to be located within the HMIS database. These data were combined with interviews to construct a final dataset that included a total of 2070 points of qualitative data and 1518 points of quantitative data. In order to answer the questions posed in this study, three of the qualitative questions were coded into 29 themes, resulting in 4002 points of data for analysis.

### 3.2. Experiences of Homelessness and Health

Participants were asked questions about their experiences with homelessness, in addition to questions about health concerns they experienced and subsequently perceived as either being a direct result of poor air quality, or as being exacerbated by poor air quality. The amount of time an individual experiences homelessness generally exacerbates health concerns [43] and is therefore a primary metric of concern. Analysis of interview data (Table 1) indicates that individuals reported experiencing an average of 138.4 weeks of time experiencing homelessness (TEH), with 50% of interviewed individuals meeting the time threshold for having chronic status (52 weeks or more TEH). For further empirical support, we used HMIS data for 130 of the overall 138 participants concerning TEH. From an institutional perspective, HMIS data indicated that individuals experienced an average of 26.4 weeks of unsheltered homelessness (HMIS TEH Unsheltered) and 26.3 weeks of sheltered homelessness (HMIS TEH Sheltered).

These results indicate a substantial difference between self-reported TEH and HMIS-reported TEH. There are limitations to both self-reported TEH and HMIS-reported TEH data. The self-reported data is an estimated time-frame based on the memory of the individual, and participants can obviously over- or underestimate these self-reports. HMIS-reported TEH only includes time points when individuals have interacted with day shelters, night shelters, outreach services, and other service providers. The limitation here is that we can accurately determine days experiencing homelessness when an individual checks in to a day or a night shelter; however, when an individual only interacts with outreach or other services, we can only estimate TEH. In order to accurately represent HMIS-reported TEH per individual, we only used the shelter instances to calculate both sheltered and unsheltered homelessness.

A variety of questions considered how IEHs participants perceived health outcomes associated with poor air quality. Health outcomes collected during interviews included self-reports of whether individuals visited a medical provider due to air pollution-related ailments (*n* = 119, 86.2%), as well as, irrespective of being associated with poor air quality, whether participants experienced difficulty breathing (*n* = 111, 80.4%), experienced headaches (*n* = 80, 58.0%), and experienced mental health illnesses (*n* = 36, 26.1%) (see Table 2). Self-reported health outcomes indicate that the majority of participants in this study had sought medical attention for air pollution-related complaints, as well as experienced difficulty breathing and headaches. Mental health outcomes were reported to affect participants to a lesser degree. Overall, these data point to high incidence of self-reported negative health outcomes.

### 3.3. Relationships between Health and Types of Homelessness

We developed statistical models to test the following hypotheses: (1) as TEH increases, negative health outcomes will be more prevalent; (2) individuals that are experiencing unsheltered homelessness will have increased negative health outcomes; and (3) individuals that are experiencing chronic homelessness will have increased negative health outcomes. Logistic regression and t-tests were used to examine the relationships between duration of homelessness, unsheltered homelessness, and chronic homelessness with health outcomes (e.g., medical visits, difficulty breathing, headache, and mental health) of research participants. Logistic regression analyses were conducted with both the self-reported TEH data and the HMIS TEH. We included gender, age, and race as control variables. The logit model used for the interview data is shown in Equation (1):

(1) $$\begin{array}{cc} {Health\;Outcome}_{i} & =\beta_{0}+\beta_{1}\;Self\;reported\;TEH_{i}+\beta_{2}\;Currently\;Sheltered_{i}+\beta_{3}\;Gender_{i}\\ & +\beta_{4}\;Age_{i}+\beta_{5}\;Race_{i} \end{array}$$

In Equations (1) and (2), and Table A1, Appendix A, $\beta_{i}\;$ stands for the unknown coefficient for each of the variables in the logistic regression analysis, which are then solved through the analysis. The results of the logistic regression analyses indicated that TEH did not significantly impact whether individuals experienced chest discomfort, headaches, mental health illness, or sought medical attention for air pollution-related illnesses. Whether individuals were currently residing in nightly shelter services or outdoors also did not affect health outcomes. Further, when subsetting the model based on chronic status, there was no difference in health outcomes or seeking medical attention for pollution-related illnesses. We were unable to explore health outcomes and seeking medical attention between individuals who were chronic and unsheltered (*n* = 51), chronic and sheltered (*n* = 18), nonchronic and unsheltered (*n* = 17), and nonchronic and sheltered (*n* = 52), as subsetting the population to these groups impacted the statistical power of the model. Additionally, using data from the statewide HMIS database, a regression equation was created to better understand the ways in which various self-reported health outcomes are affected by a combination of individuals’ time experiencing unsheltered homelessness, time experiencing sheltered homelessness, gender, age, and race. Subsequently, the logit model used for HMIS data is shown in Equation (2):

(2) $$\begin{array}{cc} Health\;Outcome{\;}_{i} & =\beta_{0}+\beta_{1}\;HMIS\;TEH\;Unsheltered_{i}\;+\;\beta_{2}\;HMIS\;TEH\;Sheltered_{i}\\ & +\beta_{3}\;Gender_{i}+\beta_{4}\;Age_{i}+\beta_{5}\;Race_{i} \end{array}$$

The results of the logistic regression analyses indicated that the duration of unsheltered homelessness (HMIS TEH unsheltered) and the duration of sheltered homelessness (HMIS TEH Sheltered) did not significantly impact whether individuals experienced chest discomfort, headaches, mental health illness, or sought medical attention for air pollution-related illnesses. When subsetting the model based on chronic status, there was no difference in health outcomes or seeking medical attention for pollution-related illnesses. T-test results were also not significant when examining whether the duration of time spent experiencing homelessness, both sheltered and unsheltered, and chronic status impacted health outcomes and seeking medical attention for pollution-related ailments of IEHs in this study. Results of the logistic regressions for health outcomes are presented in Table A1, Appendix A.

Beyond these nonsignificant inferential statistical analyses, there were a number of descriptive results from the qualitative interviews that bear substantial contribution to understanding IEH’s perspectives of environmental disamenities. The results of the thematic analyses of the qualitative data indicate that IEHs are very aware when pollution is in the air. When asked how participants were aware that pollution was in the air, 61% of respondents indicated that they experienced some kind of physical response to air pollution (Table 3). These physical responses showed that 50.4% of individuals interviewed experienced chest discomfort when air pollution is present, and 12.2% of respondents experienced ear, nose, and throat discomfort (including headaches).

When asked how air pollution impacted health, 89.1% of participants indicated that they had visited a medical professional for air pollution-related ailments (Table 4). These health-related impacts included: chest discomfort (49.6%); ear, nose, and throat discomfort, including headaches (17.9%); physical exhaustion (18.7%); and emotional stress (36.6%).

When asked about seeking professional medical attention for air pollution-related ailments, 40.6% of participants indicated that they had gone to the doctor or clinic due to air pollution health concerns (Table 5). These health-related impacts included: chest discomfort (80.4%); ear, nose, and throat discomfort, including headaches (23.2%); and emotional stress (21.4%).

A number of salient points can be determined from this in-depth examination of IEH’s reported impacts of environmental disamenities. These analyses indicate that there is no statistical difference in the health outcomes of individuals experiencing homelessness based on duration spent experiencing homelessness (chronic versus nonchronic homelessness) and whether an individual is sheltered or unsheltered. Regardless of these expected differences, the environmental hazards that this population faces results in similar levels of reported negative health outcomes.

## 4. Discussion

In addition to describing IEH’s experiences of perceived negative air quality, this study sought specifically to test hypotheses concerning individuals’ sheltered status (sheltered vs. unsheltered) and individuals’ duration of homelessness (chronic vs. nonchronic). In this section, we characterize our analyses in the context of existing literature and explain why these findings contribute novel understandings of homelessness and environment-influenced health outcomes. This study sought to understand how negative air quality experiences affected IEH’s engagements with health care providers and subjective perspectives of various health outcomes. This study provides an initial empirical inquiry to understand how environmental disamenities negatively influence IEHs, as well as noting that sheltered status and duration of homelessness are less impactful than originally hypothesized. One of the primary rationales for undertaking these research questions was that there has to date been very little empirical research on homelessness and environmental health. This dearth of research is both noteworthy and problematic given that so much of the lived experience of homelessness is spent living in and among spaces that are fundamentally affected by environmental conditions; in other words, homelessness is largely associated with being outdoors [23,26,30,44,45,46].

The descriptive results from this study provide initial understandings of how IEHs understand and characterize their health outcomes vis-a-vis an environmental disamenity. Nearly 90% of the sample indicated that they notice air pollution, with the most common way of noticing it being through sight (46.3%), followed by smell (12.3%). Further, 61% of IEHs reported having a physical reaction to air pollution and 37% of the sample reported air pollution-related emotional stress. Additionally, more than 89% of interviewees sought medical attention because of a condition associated with poor air quality. Of these participants who reported health-related pollution impacts, the majority of the concerns centered around chest complaints (49.6%), followed by exhaustion (18.7%) and ear, nose, throat, and headache complaints (17.9%). Overall, these results indicate that for people experiencing both sheltered and unsheltered homelessness in Salt Lake County, for both relatively short and extended periods of time, poor air quality is a present, often acute, corporeal, embodied, physical, and psychological experience. Such findings are novel contributions to a nascent body of literature that seeks to use an environmental justice approach to understand how a particular marginalized population—in this case, those who are facing homelessness—perceive and respond to a particular environmental disamenity (poor air quality).

Interestingly, our analyses demonstrated no significant differences in health outcomes between individuals experiencing chronic and nonchronic homelessness. An extensive amount of literature suggests that a variety of cardiopulmonary and mental health ramifications tend to occur from increased duration and incidents of exposure to negative air quality episodes [1,3,47,48,49,50,51]. However, both individual self-reports and homeless database reports indicate that individuals experiencing both nonchronic and chronic homelessness indicate negative health outcomes associated with poor air quality. Perhaps surprisingly, the absence of statistical significant difference here may indicate a variety of information about the larger population of IEHs. For instance, perhaps there are external explanatory factors about the overall homeless population that contribute to higher incidence of cardiopulmonary ill health, making differences in individuals’ duration of homelessness less than a causal factor. Statistically, there are no reported health differences associated with duration of homelessness, either sheltered or unsheltered individuals, which adds to the literature on this topic [19,52,53,54,55,56,57]. It also may be the case that even short-term experiences of homelessness contribute substantially to cardiopulmonary concerns, again negating duration as a causal, explanatory factor of differences in reported negative health outcomes.

Our analyses also demonstrated no significant difference in health outcomes between IEHs who accessed publicly available shelters and those who were unsheltered. Unsheltered homelessness generally involves sleeping outside, either in tents, abandoned buildings, or with no material shelter at all. It was therefore hypothesized that this higher duration of exposure to negative air quality episodes would increase negative health outcomes in comparison to those who access evening shelter. Again, self-reports and homeless database reports indicate that both sheltered and unsheltered homeless populations experience negative health outcomes associated with poor air quality, but there are not significant differences between these subpopulations. There are a number of interesting conjectures that emerge from these findings. For instance, one’s shelter status is rarely static. Rather, IEHs may move somewhat fluidly between sleeping in locations that are considered unsheltered and making use of publicly available shelters, depending on any number of institutional, environmental, and personal factors. It may be that imposing a binary sheltered/unsheltered status on IEHs introduces a structured distinction that has relatively little health difference. Further, the statistically nonsignificant findings may be counterintuitively encouraging for those facing unsheltered homelessness, as their nighttime exposure during poor air quality episodes does not increase their negative health experiences. Typically, and primarily due to daily automobile use patters, PM_2.5_ concentrations are higher in early morning hours, show decreases throughout the middle part of the day, and then increase again during late afternoon and into the early evening, particularly during wintertime inversion events [58,59]. These particulate concentrations then decrease overnight. Given the hourly particularities of check-in and exiting procedures at many sheltering services, it is possible that many IEHs who access nightly sheltering services might be avoiding the worst aspects of daily PM_2.5_ concentrations by being inside directly after evening rush hour commute periods, and then subsequently avoiding some of the next period of high concentrations the following morning. However, the simultaneous analysis is that for those facing sheltered homelessness, the temporary nature of nightly indoor (and usually institutionalized) sheltering does not provide substantial respite from poor air quality events. Homeless shelters often have inadequate ventilation, unhygienic bedding, and overcrowded conditions [20], and most shelters are not prepared for including individuals with higher medical needs [60]. In fact, the nonsignificant findings remind us that homelessness, in large, is experienced in public space, and often outdoors [45], where environmental exposure is felt most viscerally.

Finally, these nonstatistically significant differences between those facing sheltered and unsheltered homelessness raise a number of pressing questions for institutional responses from states, municipalities, charitable agencies, and social services providers, among others. Given these findings, what does shelter actually provide for people, beyond the bare simplistics of a bed and a place to sleep? If shelters are not providing comfort and health relief from environmental disamenities like poor air quality, what services and functions are they actually providing? Increasingly, homeless “resource centers” provide programming, employment support, and other daytime services. Perhaps shelters and resource centers cannot support IEH’s needs for environmental health, which would then further support the notion that affordable housing, as well as “housing first” policies and programs [61,62,63], is a fundamental need for this population. Environmental justice approaches to homelessness require that researchers, advocates, activists, stakeholders, and policy makers not only document and understand the spatial distribution of “environmental bads” [64], but that we also interrogate the historical and contemporary social and political systems at play that lead to disparate environmental and human health outcomes [25]. With our findings of nearly 90% of IEHs noticing air pollution and 89% seeking medical support for air pollution-related health concerns, it becomes imperative that we begin to more fully reckon with the developing proposition [23,24] that homelessness is an environmental justice concern.

## 5. Conclusions

### 5.1. Implications

This study provides initial empirical research aimed at understanding the negative impacts of environmental disamenities on IEHs. The results indicate that though the statistical analyses presented here do not show significant differences in health outcomes between individuals experiencing unsheltered v. sheltered homelessness and between individuals experiencing chronic vs. nonchronic durations of homelessness, nearly 90% of IEHs noticed air pollution and sought medical support for air pollution-related health concerns. These results highlight that sheltered and unsheltered, short- and long-term homeless populations experience negative health outcomes associated with poor air quality. Current state-led shelters and resource centers are not providing adequate protection for IEHs from environmental disamenities, specifically air pollution, and a fundamental shift towards affordable housing and “housing first” policies are required.

### 5.2. Limitations

While these early analyses illustrate some interesting findings, access to a larger data set will increase the reliability of this study. Our research plan is to gather additional survey data, in the near future, including spatial and health record data, and we have identified both environmental justice and health-focused extramural grants to fund this work. As the topic of this survey is a public health issue for individuals at higher risk for the novel coronavirus, we will also apply for a variety of fast grants for COVID-19 research relevant to this work. The data we have collected will serve as pilot data for a larger version of this study.

### 5.3. Future Work

We contend that environmental justice activist and scholarly movements should engage more deeply and systematically with experiences of homelessness, and expand our research efforts to include more environmental disamenities to understand how they are experienced differentially across housing status, both in the U.S. and elsewhere.

## Figures and Tables

**Table 1:** Summary statistics for independent variables.

Variable	*n*	Mean (SD ^1^)	Median	Min	Max
Gender (1 = male, 0 = female)	138	0.41 (0.49)	0	0	1
Age	138	46.46 (11.56)	48	19	76
Self-reported The ^2^ (weeks)	138	138.4 (226.07)	48	1	1300
HMIS ^3^ TEH unsheltered (weeks)	130	26.45 (42.39)	82.5	0	1863
HMIS TEH sheltered (weeks)	130	26.32 (47.85)	65	0	2224
HMIS TEH total (weeks)	130	54.32 (69.97)	191	0	2336
Chronic status (1 = yes, 0 = no)	138	0.5(0.5)	0.5	0	1

^1^ Standard Deviation; ^2^ Time experiencing homelessness; ^3^ Homeless Management Information System.

**Table 2:** Health outcomes experienced by IEHs in relation to air pollution.

Health Effect	Frequency	Percent
Medical visit	119	86.2
Difficulty breathing	111	80.4
Headache	80	58.0
Mental health	36	26.1

**Table 3:** Air pollution awareness and mechanisms.

Health Effect	Frequency	Percent
Notice pollution in air	123	89.1
Physical reaction	75	61.0
Chest complaint	62	50.4
Ear, nose, throat, headache complaint	15	12.2
Exhaustion	1	0.8
Nausea	1	0.8
Emotional	2	1.6
Body ache	1	0.8
Other	9	7.3
Visual	57	46.3
Taste	7	5.7
Smell	15	12.2
Air quality alerts	2	1.6

**Table 4:** Health-related pollution impacts.

Health Effect	Frequency	Percent
Pollution related doctor visit	123	89.1
Chest complaint	61	49.6
Ear, nose, throat, headache complaint	22	17.9
Exhaustion	23	18.7
Nausea	8	6.5
Emotional	45	36.6
Body ache	4	3.3
Other	22	17.9

**Table 5:** Reasons for seeking medical attention for pollution-related health effects.

Health Effect	Frequency	Percent
Pollution related doctor visit	56	40.6
Chest complaint	45	80.4
Ear, nose, throat, headache complaint	13	23.2
Exhaustion	5	8.9
Nausea	2	3.6
Emotional	12	21.4
Body ache	1	1.8
Other	7	12.5

## Appendix A

**Table A1:** Logistic regression results for health outcomes.

Health Outcome	Variables	𝛽 ^1^	Standard Error	Pr(>|z|) ^2^
**Medical Visit**				
	Intercept	−2.40	1.43	0.0932
	TEH	0.00	0.00	0.2649
	Currently Sheltered	0.29	0.60	0.6246
	Gender	0.38	0.57	0.5004
	Age	0.07	0.03	0.0115
	Race—Native American or Alaskan American	17.22	1817.00	0.9924
	Race—Asian	−0.05	1.45	0.9746
	Race—Black or African American	0.55	1.16	0.6343
	Race—Refused	17.46	6523.00	0.9979
	Race—Native Hawaiian or Other Pacific Islander	16.17	6523.00	0.998
	Race—White	0.83	0.91	0.3613
**Difficulty Breathing**				
	Intercept	0.08	1.20	0.9458
	TEH	0.00	0.00	0.0944
	Currently Sheltered	−0.89	0.55	0.1093
	Gender	0.48	0.48	0.3236
	Age	0.02	0.02	0.4176
	Race—Native American or Alaskan American	0.68	1.02	0.5071
	Race—Asian	0.12	1.23	0.9243
	Race—Black or African American	0.99	1.02	0.3355
	Race—Refused	14.89	1455.00	0.9918
	Race—Native Hawaiian or Other Pacific Islander	15.31	1455.00	0.9916
	Race—White	1.34	0.83	0.1059
**Headache**				
	Intercept	0.45	1.07	0.675
	TEH	0.00	0.00	0.108
	Currently Sheltered	−0.34	0.46	0.457
	Gender	0.16	0.41	0.698
	Age	0.01	0.02	0.688
	Race—Native American or Alaskan American	1.23	1.07	0.25
	Race—Asian	−0.09	1.19	0.937
	Race—Black or African American	0.44	0.96	0.647
	Race—Refused	15.13	1455.00	0.992
	Race—Native Hawaiian or Other Pacific Islander	15.03	1455.00	0.992
	Race—White	0.35	0.78	0.65
**Mental Health**				
	Intercept	−18.38	2517.00	0.9942
	TEH	0.00	0.00	0.7634
	Currently Sheltered	−0.30	0.76	0.6941
	Gender	1.14	0.65	0.0808
	Age	−0.01	0.03	0.5876
	Race—Native American or Alaskan American	18.22	2517.00	0.9942
	Race—Asian	0.63	5249.00	0.9999
	Race—Black or African American	17.89	2517.00	0.9943
	Race—Refused	−0.91	6992.00	0.9999
	Race—Native Hawaiian or Other Pacific Islander	18.41	2517.00	0.9942
	Intercept	1.34	0.83	0.1059
**Medical Visit**				
	Intercept	14.90	6523.00	0.9982
	HMIS TEH Unsheltered	0.00	0.01	0.5797
	HMIS TEH Sheltered	0.01	0.01	0.3211
	gender	0.55	0.61	0.3637
	age	0.06	0.03	0.0198
	Race—Native American or Alaskan American	0.09	6766.00	1
	Race—Asian	−17.21	6523.00	0.9979
	Race—Black or African American	−16.53	6523.00	0.998
	Race—Native Hawaiian or Other Pacific Islander	−0.60	9224.00	0.9999
	Race—White	−16.33	6523.00	0.998
**Difficulty Breathing**				
	Intercept	15.94	2400.00	0.9947
	HMIS TEH Unsheltered	0.00	0.00	0.3667
	HMIS TEH Sheltered	−0.01	0.00	0.0774
	gender	0.25	0.51	0.6274
	age	0.01	0.02	0.6424
	Race—Native American or Alaskan American	−14.94	2400.00	0.995
	Race—Asian	−15.71	2400.00	0.9948
	Race—Black or African American	−14.84	2400.00	0.9951
	Race—Native Hawaiian or Other Pacific Islander	0.01	3393.00	1
	Race—White	−14.49	2400.00	0.9952
**Headache**				
	Intercept	15.61	1455.00	0.9914
	HMIS TEH Unsheltered	−0.01	0.00	0.0896
	HMIS TEH Sheltered	−0.01	0.00	0.1098
	gender	−0.02	0.43	0.9707
	age	0.00	0.02	0.9126
	Race—Native American or Alaskan American	−13.39	1455.00	0.9927
	Race—Asian	−14.84	1455.00	0.9919
	Race—Black or African American	−14.41	1455.00	0.9921
	Race—Native Hawaiian or Other Pacific Islander	−0.14	2058.00	0.9999
	Race—White	−14.49	1455.00	0.9921
**Mental Health**				
	Intercept	−17.07	3956.00	0.9966
	HMIS TEH Unsheltered	0.00	0.00	0.2663
	HMIS TEH Sheltered	0.01	0.01	0.1781
	gender	0.97	0.44	0.0287
	age	−0.03	0.02	0.0766
	Race—Native American or Alaskan American	17.05	3956.00	0.9966
	Race—Asian	0.36	4312.00	0.9999
	Race—Black or African American	16.06	3956.00	0.9968
	Race—Native Hawaiian or Other Pacific Islander	1.65	5595.00	0.9998
	Race—White	17.00	3956.00	0.9966

^1^ Solved coefficient for each of the variables in the logistic regression analysis; ^2^ Two-tailed significance.
